# Effects of a Tailored Brief Behavioral Therapy Application on Insomnia Severity and Social Disabilities Among Workers With Insomnia in Japan: A Randomized Clinical Trial

**DOI:** 10.1001/jamanetworkopen.2020.2775

**Published:** 2020-04-14

**Authors:** Isa Okajima, Jou Akitomi, Ikuo Kajiyama, Miho Ishii, Hiroto Murakami, Mineko Yamaguchi

**Affiliations:** 1 Department of Psychological Counseling, Faculty of Humanities, Tokyo Kasei University, Tokyo, Japan; 2 NEC Solution Innovators Ltd, Tokyo, Japan; 3 Senzoku Stress Coping Support Office, Tokyo, Japan; 4 NEC VALWAY Ltd, Tokyo, Japan

## Abstract

**Importance:**

According to the stepped-care model, there is a medium to large effect size for using cognitive behavioral therapy for insomnia that is delivered digitally, such as a smartphone application. However, it has been reported that studies using fully automated cognitive behavioral therapy for insomnia applications without expert support have high dropout rates.

**Objective:**

To examine the effects of using a fully automated and individually tailored brief behavior therapy for insomnia (BBTI) applications for 2 weeks on insomnia-related symptoms, social disabilities, and work productivity among workers with insomnia in Japan.

**Design, Setting, and Participants:**

This intent-to-treat prospective parallel-group randomized clinical trial included participants 20 years or older with Insomnia Severity Index (ISI) scores of 8 or higher. Participants were recruited via internet advertisements and workplace flyers and randomized to tailored BBTI, standard BBTI, self-monitoring with sleep diaries, or a waiting list control group. The study was conducted from September 21, 2017, to February 23, 2018. Data were analyzed from February 24, 2018, to February 22, 2019.

**Interventions:**

A personalized BBTI and standard BBTI intervention, both of which included sleep scheduling, relaxation, sleep hygiene, and sleep diaries, administered via smartphone application.

**Main Outcomes and Measures:**

Primary outcomes were insomnia severity, measured using the Japanese version of the Insomnia Severity Index, and social disabilities, measured using the Japanese version of the Sheehan Disability Scale. Secondary outcomes were dysfunctional beliefs, sleep reactivity, and work productivity. All measures were taken before and after the intervention and at 1-month and 3-month follow-ups.

**Results:**

A total of 92 participants (mean [SD] age, 42.7 [11.5] years; 60 [65%] men) were randomized and included in analysis, with 24 participants assigned to tailored BBTI, 23 participants assigned to standard BBTI, 23 participants assigned to self-monitoring, and 22 participants assigned to the waiting list control group. At baseline, there were no significant differences among groups on any demographic characteristics or outcome measures. The results of the intent-to-treat analysis showed an interaction effect for all outcome measures. Compared with the waiting list control group, the BBTI interventions were more effective for reduction of insomnia severity (tailored BBTI: Hedges *g* = –1.64 [95% CI, –2.32 to –0.96]; *P* < .001; standard BBTI: *g* = –1.28 [95% CI, –1.93 to –0.63]; *P* < .001), social disabilities relating to social life (tailored BBTI: *g* = –1.33 [95% CI, –1.97 to –0.68]; *P* < .001; standard BBTI: *g* = –0.84 [95% CI, –1.46 to –0.22]; *P* = .009), and dysfunctional beliefs (tailored BBTI: *g* = –1.17 [95% CI, –1.80 to –0.54]; *P* < .001; standard BBTI: *g* = –0.84 [95% CI, –1.46 to –0.23]; *P* = .02) at the 3-month follow-up. Tailored BBTI quickly reduced insomnia severity (1-month follow-up: *g* = –0.85 [95% CI, –1.46 to –0.24]). Tailored BBTI was only more effective for improvement of work performance (*g* = –1.09 [95% CI, –1.71 to –0.46]; *P* = .005), social disabilities related to family life (g = –0.89 [95% CI, –1.51 to –0.28]; *P* = .005), and sleep reactivity (*g* = –1.09 [95% CI, –1.72 to –0.46]; *P* = .007) compared with the waiting list control group at the 3-month follow-up. The tailored BBTI led to improved worker productivity compared with standard BBTI (g = 0.94 [95% CI, 0.33 to 1.55]; *P* = .01) at the 3-month follow-up.

**Conclusions and Relevance:**

These findings suggest that an application for individually tailored BBTI is an inexpensive and effective treatment for insomnia. In future research, it would be informative to investigate the reasons for dropout during the follow-up period.

**Trial Registration:**

umin.ac.jp/ctr Identifier: UMIN000036572

## Key Points

**Question:**

Does a smartphone application for brief behavioral therapy for insomnia improve insomnia-related symptoms and worker productivity?

**Findings:**

In this randomized clinical trial including 92 workers with insomnia, use of an application that provided tailored brief behavioral therapy for insomnia for 2 weeks significantly decreased insomnia severity and social disabilities and improved work performance after 3 months.

**Meaning:**

These findings suggest that individually tailored brief behavioral therapy for insomnia delivered via smartphone application is an inexpensive and effective treatment for workers with insomnia.

## Teaser

This randomized clinical trial examines the effects of tailored brief behavioral therapy delivered via smartphone application on insomnia severity and social disabilities among workers in Japan.

## Introduction

Nearly 20% of the general adult population is reported to have experienced symptoms of insomnia.^1,2,3^ Additionally, 10% to 15% of people with insomnia experience chronic insomnia,^1,4,5^ which is associated with the development or relapse of depression, as well as resistance to depression treatment.^6,7^ Cognitive behavioral therapy (CBT) for insomnia (CBTI) has been recommended as an effective first choice intervention for chronic insomnia disorder,^8,9^ and it has been found to be effective for improving insomnia symptoms in 70% to 80% of patients^10^ and to have a long-term preventive effect on symptom recurrence.^11^ Meta-analyses indicate that CBTI has moderate to large lasting effects on insomnia severity, sleep quality, sleep efficiency, sleep-onset latency, and wake-up time after sleep onset.^12,13,14^

In general, a full course of CBTI is conducted face-to-face and carried out during 4 to 8 weekly sessions, while brief behavioral therapy for insomnia (BBTI) can be carried out in less than 4 weeks in a clinical or primary care setting.^15,16^ However, it may be difficult to schedule more than a few appointments with a health care practitioner (eg, physician, clinical psychologist, or nurse) who is also responsible for the care of hundreds of other patients. Since there are many individuals with insomnia symptoms,^3^ a face-to-face intervention may not be feasible treatment for individuals with insomnia who need CBTI.

In a 2011 proposal by Mack and Rybarczyk,^17^ a stepped-care model of psychological management for insomnia was suggested. A stepped-care model can be thought of as a pyramid. The model by Mack and Rybarczyk^17^ proposes attempting lower-cost interventions as the first choice, while more expensive and intensive interventions are reserved for individuals who do not respond to less intensive interventions. The least intensive interventions are self-help courses, such as bibliotherapy and internet- or application-delivered treatments. These interventions are less expensive and potentially more readily available to a greater number of people with insomnia. These interventions are also used to alleviate more insomnia symptoms at the bottom of the pyramid, while fewer individuals with insomnia who do not respond to these treatments receive progressively more intensive and individualized treatments toward the top of the pyramid. A meta-analysis that analyzed computerized CBTI reported relative success with a medium to large effect size.^18^

Many studies into the efficacy of digitally delivered CBTI have had experimental limitations that may have affected the outcomes. First, the insomnia remission rate for CBTI with psychological support from CBT experts has been reported as higher than that of CBTI without psychological support (61% vs 24%).^19^ The low remission rate associated with CBTI without psychological support may be associated with its use in patients with more severe chronic insomnia disorder. These individuals are likely to require more intensive support from higher levels in the pyramid, such as support from a CBT expert. On the other hand, it is necessary to enhance the effect of digitally delivered CBTI without additional expert support when the intervention is provided for a greater number of individuals with insomnia at the bottom of the pyramid. Studies have shown that individuals who use fully automated CBTI applications for 6 to 8 weeks of treatment without expert support have better improvements in insomnia severity and sleep-related quality of life than control groups.^20,21^ However, despite being highly effective, the second limitation of fully automated CBTI studies is the dropout rate, which has been reported as 39% to 45%, higher than that of face-to-face CBTI.^20,21^ Therefore, the number of sessions that patients complete could be a crucial factor for the success of fully automated CBTI as an intervention.

According to the stepped-care model, providing CBTI to people with insomnia symptoms is an important preventive measure for first- and second-stage interventions for individuals with less severe insomnia. Vincent and Walsh^22^ examined the factors associated with a patient’s movement through a stepped-care pathway using 50 adults with chronic insomnia. They found that digitally delivered CBTI was sufficient to improve insomnia symptoms particularly for younger adults, individuals who were employed, and individuals with less severe insomnia.

For the treatment of depression, individually tailored, internet-delivered CBT has been shown to be more effective for many participants than a standardized intervention.^23^ Additionally, Forsell et al^24^ reported that individually tailored interventions reduced the number of failed treatments, although it is worth noting that patients in that study also had time with a therapist.

However, to our knowledge, no study of digitally delivered CBTI has made a direct comparison between tailored and standardized interventions that takes into consideration how these differ from waiting list controls. To approach this problem, we developed a smartphone application that provides a 2-week tailored BBTI designed to enhance the effect of digitally delivered CBTI and to reduce the dropout rate during the intervention. The aim of this study was to examine the effects of a fully automated and individually tailored intervention on insomnia-related symptoms, social disabilities, and worker productivity among workers with insomnia compared with standard BBTI, self-monitoring, and a waiting list control group.

## Methods

This study was approved by the Ethics Committee of the Waseda University. All participants provided written informed consent. The study was conducted and reported in accordance with the Consolidated Standards of Reporting Trials (CONSORT) reporting guidelines.

### Participants

**Figure 1.**
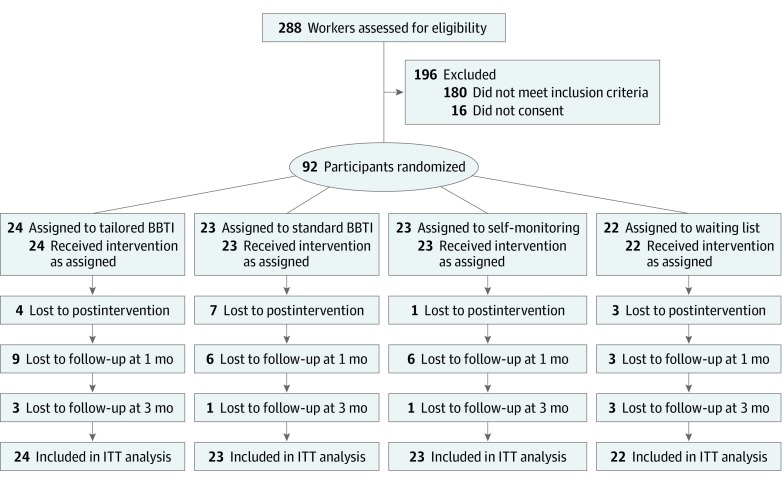
Study Flowchart BBTI indicates brief behavioral therapy for insomnia and ITT, intention-to-treat.

We recruited participants from September 21 to October 11, 2017, through websites and flyer distribution to companies in Japan. Workers who entered the study completed an informed consent form and started the online questionnaires. Inclusion criteria were (1) workers 20 years or older, (2) total score in the Insomnia Severity Index (ISI) of 8 or higher, (3) reporting initial, middle, or terminal insomnia, and (4) performing work during the day. Exclusion criteria were (1) having a medical history of posttraumatic stress disorder, depressive disorders, or sleep disorders without insomnia, (2) reporting suicidal ideation, (3) receiving pharmacological or psychological treatments, (4) working during non-daytime hours, and (5) being at high risk of causing serious harm from sleep loss (eg, individuals who operate heavy machinery) (Figure 1). The trial began on September 21, 2017, and the study completion date, including the follow-up period, was February 23, 2018. Data analysis was conducted from February 24, 2018, to February 22, 2019.

### Study Design

This randomized clinical trial used a prospective parallel-group design, and investigators and assessors were blinded and individually randomized. An independent system engineer generated random allocation sequences using a computer and stratified randomization by age and sex. Therefore, all authors were blind to the allocation. Participants were randomized to tailored BBTI, standard BBTI, self-monitoring with sleep diaries, or waiting list control.

For the purpose of informed consent, participants received information on background and purpose of the research, type of research intervention, voluntary participation, duration, risks, benefits, confidentiality, sharing the results, right to refuse or withdraw, alternative to participating, and who to contact. If they agreed to all contents, they were asked to sign an agreement form.

### Assessment Measures

Participants were assessed via an internet form at baseline, 2 weeks after the intervention, and at the 1-month and 3-month follow-ups. The primary outcomes were insomnia severity, measured using the total score of the Japanese version of ISI,^25^ and social disability, measured using the Japanese version of Sheehan Disability Scale (SDISS).^26^ The ISI consists of 7 items assessing insomnia severity, and the SDISS consists of 3 items assessing disabilities in work performance, social life, and family life. The instructions of each subscale of the SDISS were modified from *The symptoms have disrupted* to *Your sleep problems have disrupted* (in Japanese).

The secondary outcomes were scores on the 16-item Japanese version of Dysfunctional Beliefs and Attitudes about Sleep scale (DBAS-16),^27^ the Japanese version of Ford Insomnia Response to Stress Test (FIRST),^28^ and the Japanese version of Work Limitation Questionnaire (WLQ).^29^ The DBAS-16 measures dysfunctional thoughts about sleep, the FIRST equivalent consists of 9 items and measures sleep reactivity to stress (ie, hyperarousal), and the WLQ consists of 25 items and measures work productivity affected by presenteeism (ie, low performance at work transformed to lost workday equivalents).

### Sample Size

Sample size was based on a power analysis conducted for the ISI scores. Effect sizes were estimated from 2 weeks of BBTI pilot data acquired by our group prior to this study: Hedges *g* in the mean (SD) ISI scores from baseline (mean [SD], 12.38 [3.57]) to 1-month follow-up (mean [SD], 8.48 [3.41]) was 1.10 in 21 workers with insomnia. With power of 0.9 to detect a significant difference at *P* = .05 (2-sided), it was calculated that 12 individuals would be required for each group. Since the reported dropout rate for self-help CBTI is highly variable among studies,^30^ we aimed to allow for a 40% dropout rate, which required 20 participants to be recruited per group.

### Interventions

Participants downloaded the application for sleep improvement developed for this study to their Android or iPhone smartphone. All participants were assessed for sleep-related daily habits (eg, bed/wake time, physical activities, exposure to bright light) before the intervention, 2 weeks after the intervention, and at the 1-month and 3-month follow-ups. Each participant was randomized to 1 of 4 groups: tailored BBTI, standard BBTI, self-monitoring, or waiting list (control group). Full details on interventions are provided in the trial protocol in Supplement 1.

#### Tailored BBTI

For participants in the tailored BBTI group, we prepared 26 challenge tasks based on the results of their baseline assessments (eTable 1 in Supplement 2). The challenge tasks were developed based on techniques for stimulus control (eg, getting out of bed when unable to sleep or going to bed only when sleepy), sleep restriction (eg, setting a regular sleep window for going to bed and getting out of bed), relaxation (eg, conducting breathing relaxation before sleep every night), and sleep hygiene (eg, taking time outside in the morning, such as a walk, for sunlight exposure). For each challenge task, the difficulty and effect levels ranged from 1 star (ie, very easy or low effect) to 5 stars (ie, very difficult or high effect). For example, an individual who reported difficulty initially getting to sleep would be suggested the challenge task *get out of bed when unable to sleep and go to bed only when sleepy* based on the stimulus control technique (difficulty level: 5 stars; effect level: 5 stars). Alternatively, for an individual who reported difficulty maintaining sleep would be suggested the challenge task *set a regular sleep window* (difficulty level: 5 stars; effect level: 5 stars) based on the sleep restriction technique.

Participants chose 1 to 3 of the tasks that had been suggested to them, then they focused on these tasks for 2 weeks. Each day, they reported whether each task was implemented and recorded sleep conditions, including bed and wake times, sleep onset latency, waking after sleep onset, napping, and vitality during the day, using a sleep diary. Messages were sent through the application to participants each day during the 2 weeks to remind them to keep recording the sleep diary. When the task was not conducted often enough, instructions to rethink the task were presented with several specific examples on the 3rd and 10th day. For example, if participants found it difficult to conduct the challenge task *get out of bed when unable to sleep and go to bed only when sleepy*, they were provided with additional suggestions, such as not checking the time if they awoke in the night. In addition, titrating time in bed was performed based on whether a participant reported that vitality during the day was affected by sleep condition. To motivate participants to conduct the challenge tasks, a picture of the face of one of the authors (I.O.), an expert in sleep science, was presented to provide instructions and feedback comments. Additionally, an article about sleep sciences (eg, about the relationship between the circadian rhythm and exposure to bright lights) was delivered every day along with individually tailored challenge tasks.

#### Standard BBTI

Participants assigned to the standard BBTI group were given a standard set of tasks relating to sleep hygiene for week 1. They could view articles about sleep science (eg, the mechanisms of sleep homeostasis and circadian rhythms) at any time, although only 1 article about sleep science was delivered each day. For week 2, articles about sleep scheduling and relaxation techniques were delivered, and some related tasks were suggested. Participants were encouraged to pursue all suggested tasks, and they recorded a sleep diary for 2 weeks. On the 7th and 14th days, participants were given feedback on sleep and implementation status.

#### Self-monitoring

Participants assigned to the self-monitoring group recorded a sleep diary for 2 weeks. In addition, they could view all articles about sleep science at any time, although only 1 column about sleep science was delivered each day. After completing the questionnaires at the 3-month follow-up, participants were allowed to enroll in a program of tailored BBTI.

#### Waiting List

Participants assigned to the waiting list control group were only asked to complete questionnaires at baseline, 2 weeks after the intervention, and at the 1-month and 3-month follow-ups. After completing the questionnaires at the 3-month follow-up, participants were allowed to enroll in a program of tailored BBTI.

### Statistical Analysis

Most analyses were based on the intent-to-treat model. To examine the effect of tailored BBTI on insomnia-related symptoms and productivity, a generalized linear model, which compensates for missing data, was used to compare preintervention, postintervention, 1-month follow-up, and 3-month follow-up data in all groups. If data were missing, they were compensated through the multiple imputation method using Markov chain Monte Carlo estimation because it is significant at Little missing completely at random test (χ^2^_27_ = 41.75; *P* = .04). The multiple imputation used age, sex, and variables measured at each period for each group by the multivariate imputation by chained equations algorithm. When main or interaction effects in all analyses were shown, we performed Bonferroni-Holm correction for *P* values, then conducted post hoc analyses. We introduced the Bonferroni-Holm correction for *P* values, which provided more conservative *P* value estimations, to avoid the risk to observing significant *P* values because of repeated post hoc analyses. Descriptive statistics were computed using R statistical software version 3.4.4 (R Project for Statistical Computing).

In addition, we estimated the effect sizes of scales within and between groups by using correcting biases for Hedges *g*. In general, an absolute *g* value of 0.2 or more indicates a small effect size; approximately 0.5, moderate; and 0.8 or more, large.^31^ The effect sizes of all scales within groups were computed as baseline vs after the intervention, baseline vs 1-month follow-up, and baseline vs 3-month follow-up. Effect sizes between groups were analyzed after the intervention, at the 1-month follow-up, and at the 3-month follow-up. For all scales, except for the WLQ, the more negative the change in the effect size, the larger the therapeutic effect.

To compare dropout rates among groups, we conducted χ^2^ tests. Also, we conducted probit regression analysis by the number of participants who dropped out in each period as the dependent variable, and group and ISI at baseline, postintervention period, or 1-month follow-up as independent variables.

## Results

### Baseline Characteristics

A total of 288 individuals responded to our recruitment materials, and 196 individuals (68%) were excluded because they did not meet inclusion criteria or did not consent to participate. A total of 92 participants (mean [SD] age, 42.7 [11.5] years; 60 [65%] men) were included in the study and randomized, including 24 participants randomized to tailored BBTI, 23 participants randomized to standard BBTI, 23 participants randomized to self-monitoring, and 22 participants randomized to the waiting list. Demographic characteristics for each group are presented in the Table, and descriptive statistics of all measures for each group at baseline, after the intervention, 1-month follow-up, and 3-month follow-up are presented in eTable 2 in Supplement 2. At baseline, there were no significant differences among groups on any demographic characteristics or outcome measures.

**Table.** Demographic Characteristics in Each Group

Characteristic	No. (%)			
	Brief behavioral therapy for insomnia		Self-monitoring (n = 23)	Waiting list (n = 22)
	Tailored (n = 24)	Standard (n = 23)		
Age, mean (SD), y	43.4 (11.9)	42.6 (11.3)	43.9 (11.3)	40.7 (10.9)
Men	14 (58)	16 (70)	16 (70)	14 (64)
Occupation				
Office work	22 (92)	20 (87)	23 (100)	21 (96)
Specialist job	0	1 (4)	0	0
Freelance	1 (4)	1 (4)	0	0
Part-time job	1 (4)	1 (4)	0	1 (5)

There were no significant differences in the dropout rates among groups at 2 weeks after the intervention (tailored BBTI: 4 participants [17%]; standard BBTI: 7 participants [30%]; self-monitoring: 1 participant [4%]; waiting list: 3 participants [14%]), at the 1-month follow-up (tailored BBTI: 9 participants [38%]; standard BBTI: 6 participants [26%]; self-monitoring: 6 participants [26%]; waiting list: 3 participants [14%]), or at the 3-month follow-up (tailored BBTI: 3 participants [13%]; standard BBTI: 1 participant [4%]; self-monitoring: 1 participant [4%]; waiting list: 3 participants [14%]) (χ^2^_6_ = 2.33; *P* = .89). In the probit regression analysis, there were no significant effects associated with the number of individuals who dropped out. None of the participants reported any serious adverse events.

### Insomnia Severity

**Figure 2.**
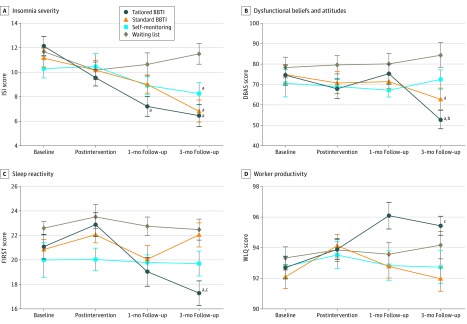
Changes in Outcome Measures Through Time for Each Group Error bars indicate SE; BBTI, brief behavioral therapy for insomnia; DBAS, Dysfunctional Beliefs and Attitudes about Sleep; and WLQ, Work Limitation Questionnaire. ^a^Statistically significantly different compared with the waiting list group. ^b^Statistically significantly different compared with the self-monitoring group. ^c^Statistically significantly different compared with the standard BBTI group.

We found a significant effect of time (*F*_3,88_ = 25.48; *P* < .001; η_G_^2^ = 0.09) and interaction (*F*_9,264_ = 5.25; *P* < .001; η_G_^2^ = 0.06) for the total ISI score (Figure 2). In the post hoc analysis, the tailored BBTI group showed significantly more improvement in ISI score than the waiting list group at the 1-month follow-up (g = –0.85 [95% CI, –1.46 to –0.24]; *P* = .04). At the 3-month follow-up, ISI scores showed significantly more improvement vs the waiting list group for individuals in the tailored BBTI (g = –1.64 [95% CI, –2.32 to –0.96]; *P* < .001), standard BBTI (g = –1.28 [95% CI, –1.93 to –0.63]; *P* < .001), and self-monitoring (g = –0.75 [95% CI, –1.36 to –0.13]; *P* = .01) groups.

For within-group comparisons, individuals in the tailored BBTI group had significantly lower mean (SD) ISI scores compared with baseline (12.13 [3.80]) after the intervention (9.54 [3.68]; *P* = .02), at the 1-month follow-up, (7.21 [3.71]; *P* < .001), and at the 3-month follow-up (6.46 [1.69]; *P* < .001). Individuals in the standard BBTI group also had a statistically significantly lower mean (SD) ISI score compared with baseline (11.17 [3.19]) at the 3-month follow-up (6.83 [3.13]; *P* < .001). Similarly, individuals in the self-monitoring group had a lower mean (SD) ISI score compared with baseline (10.30 [3.88]) at the 3-month follow-up (8.26 [4.50]; *P* = .05). Full within-group comparisons for all outcomes are presented in eTable 2 in Supplement 2.

### Social Disabilities

The results of the analysis of the all subscales of SDISS showed a significant effect of time (social life: *F*_3,88_ = 7.57; *P* < .001; η_G_^2^ = 0.03; work performance: *F*_3,88_ = 8.94; *P* < .001; η_G_^2^ = 0.04; family life: *F*_3,88_ = 3.67; *P* = .01; η_G_^2^ = 0.02). Furthermore, there were statistically significant interaction effects in SDISS social life scores (*F*_9,264_ = 6.03; *P* < .001; η_G_^2^ = 0.07) and work performance scores (*F*_9,264_ = 3.31; *P* < .001; η_G_^2^ = 0.04), but scores for the SDISS family life subscale showed no statistically significant interaction effect (*F*_9,264_ = 1.74; *P* = .08; η_G_^2^ = 0.02).

Post hoc analyses showed that the SDISS social life score for the tailored BBTI group at the 3-month follow-up was statistically significantly improved compared with the self-monitoring group (*g* = –1.14 [95% CI, –1.77 to –0.52]; *P* = .003) and waiting list group (*g* = –1.33 [95% CI, –1.97 to –0.68]; *P* = .009). Similarly, the SDISS social life score for the standard BBTI group at the 3-month follow-up was statistically significantly improved compared with the waiting list group (g = –0.84 [95% CI, –1.46 to –0.22]; *P* = .009) (eFigure 1 in Supplement 2). In within-group analyses for the tailored BBTI group, mean (SD) social life scores compared with baseline (2.75 [2.15]) were statistically significantly lower at the 1-month follow-up (1.83 [1.55]; *P* = .04) and 3-month-follow-up (0.58 [1.10]; *P* = .001). Among the standard BBTI group, mean (SD) social life scores compared with baseline 3.09 (2.17) were significantly lower at the 1-month follow-up (1.57 [1.78]; *P* = .03) and 3-month follow-up (1.39 [1.62]; *P* = .004). Furthermore, mean (SD) social life scores for individuals in the waiting list control group increased statistically significantly from 2 weeks after the intervention (2.00 [2.18]) to the 3-month follow-up (3.23 [2.60]; *P* = .04) (eTable 2 in Supplement 2).

Post hoc analyses showed that SDISS work performance scores were statistically significantly improved in the tailored BBTI group at the 3-month follow-up compared with the waiting list group (*g* = –1.09 [95% CI, –1.71 to –0.46]; *P* = .005) (eFigure 1 in Supplement 2). Furthermore, within-group analyses in the tailored BBTI group found that mean (SD) social life scores were statistically significantly reduced from baseline (3.04 [2.03]) to the 3-month follow-up (1.25 [1.11]; *P* < .001).

In post hoc analyses of SDISS family life subscale scores, the tailored BBTI group had statistically significantly improved scores at 3-month follow-up compared with the waiting list group (*g* = –0.89 [95% CI, –1.51 to –0.28]; *P* = .005) (eFigure 1 in Supplement 2). In addition, individuals in the tailored BBTI group had statistically significantly lower mean (SD) family life scores from baseline (1.67 [1.69]) to the 3-month follow-up (0.46 [0.66]; *P* = .02) (eTable 2 in Supplement 2).

### Dysfunctional Beliefs and Sleep Reactivity

Analysis for the DBAS-16 and FIRST scores showed a significant effect of time (DBAS-16: *F*_3,88_ = 3.46; *P* = .02; η_G_^2^ = 0.01; FIRST: *F*_3,88_ = 6.12; *P* = .001; η_G_^2^ = 0.02) and interaction (DBAS-16: *F*_9,264_ = 4.23; *P* < .001; η_G_^2^ = 0.04; FIRST: *F*_9,264_ = 3.18; *P* = .001; η_G_^2^ = 0.04). Post hoc analyses found that the DBAS-16 score in the tailored BBTI group at the 3-month follow-up was statistically significantly improved compared with the self-monitoring group (g = –0.79 [95% CI, –1.39 to –0.19]; *P* = .03) and waiting list group (g = –1.17 [95% CI, –1.80 to –0.54]; *P* < .001). Similarly, DBAS-16 score in the standard BBTI group at the 3-month follow-up was significantly improved compared with the waiting list group (g = –0.84 [95% CI, –1.46 to –0.23], *P* = .02) (Figure 2). Within-group analyses in the tailored BBTI group found statistically significantly reduced mean (SD) DBAS-16 score from baseline (74.63 [24.29]) to the 3-month follow-up (52.83 [24.82]; *P* = .01). For within-group analysis of the standard BBTI, mean (SD) DBAS-16 score was statistically significantly reduced from baseline (74.96 [23.02]) to the 3-month follow-up (62.91 [21.48]; *P* = .01).

Post hoc tests showed that the tailored BBTI group had statistically significantly improved FIRST score at the 3-month follow-up compared with the standard BBTI group (*g* = –1.02 [95% CI, –1.63 to –0.40]; *P* = .004) and waiting list group (*g* = –1.09 [95% CI, –1.72 to –0.46]; *P* = .007) (Figure 2). In addition, within-group analyses found a statistically significantly difference in mean (SD) FIRST scores in the tailored BBTI group from baseline (21.08 [4.94]) to the 3-month follow-up (17.29 [3.18]; *P* = .004).

### Work Productivity

Analysis of the WLQ results showed a significant effect of time (*F*_3,88_ = 3.99; *P* = .008; η_G_^2^ = 0.01) and interaction (*F*_9,264_ = 3.03; *P* = .002; η_G_^2^ = 0.03). Post hoc tests showed that the WLQ score in the tailored BBTI group at the 3-month follow-up was significantly improved compared with the standard BBTI group (*g* = 0.94 [95% CI, 0.33 to 1.55]; *P* = .01) (Figure 2). In addition, individuals in the tailored BBTI group had statistically significantly different mean (SD) WLQ scores compared with baseline (92.70 [3.63]) at the 1-month follow-up (96.09 [3.37]; *P* = .001) and 3-month follow-up (95.43 [3.72]; *P* = .003). In the standard BBTI group, mean (SD) WLQ score at baseline (92.12 [3.14]) was statistically significantly different 2 weeks after the intervention (94.10 [3.70]; *P* = .02).

**Figure 3.**
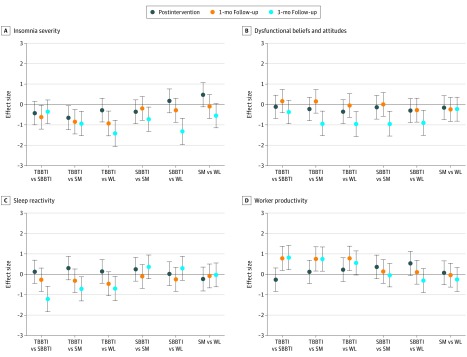
Plots of Effect Sizes Through Time for Each Outcome Measure Between Groups Error bars indicate 95% CI; SBBTI, standard brief behavioral therapy for insomnia; SM, self-monitoring; TBBTI, tailored brief behavioral therapy for insomnia; and WL, waiting list.

**Figure 4.**
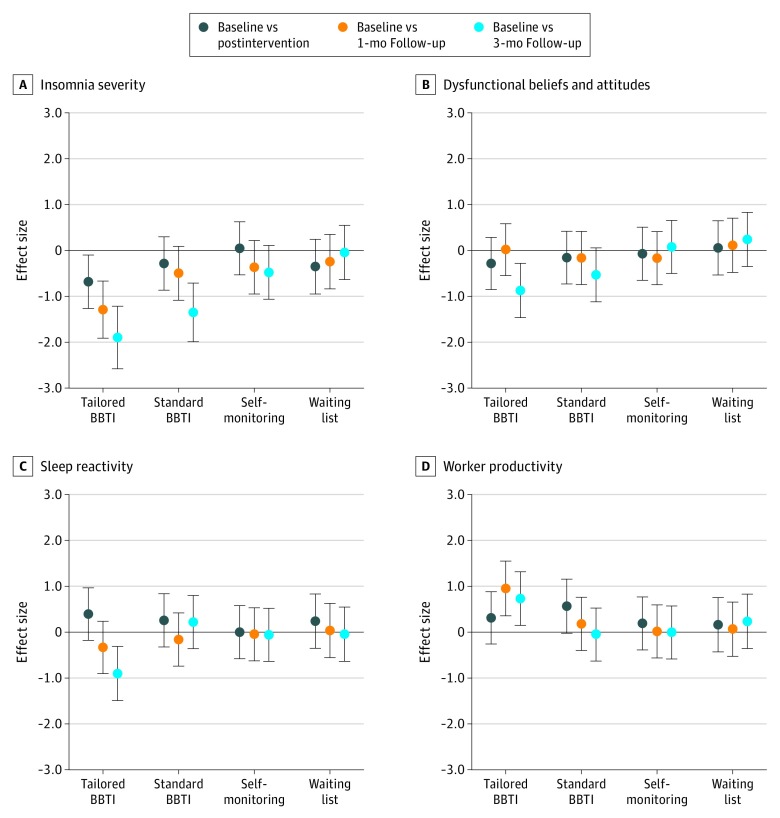
Plots of Effect Sizes (Hedges *g*) for Each Outcome Measure Within Each Group Error bars indicate 95% CI and BBTI, brief behavioral therapy for insomnia.

The effect sizes in ISI, DBAS-16, FIRST, and WLQ scores between and within groups are presented in Figure 3 and Figure 4. Effect sizes in SDISS scores between and within groups are presented in eFigure 2 and eFigure 3 in Supplement 2.

## Discussion

This randomized clinical trial examined the effects of a smartphone application of tailored BBTI for workers with insomnia on insomnia severity, social disabilities, and work productivity. The mean age of participants was 42.7 years, which corresponds to a relatively young group of employees. A review by Morin et al^10^ reported that older patients are less responsive to behavioral treatments than middle-aged or younger adults. In addition, CBTI that uses digital technology has been reported as particularly effective for younger adults and employees.^22^ Therefore, providing BBTI for participants is appropriate according to a stepped-care model for insomnia. After the intervention, the dropout rate for tailored BBTI was lower than that for standard BBTI (17% vs 30%), although the difference was not statistically significant. This means that the participants who received standard BBTI dropped out of the intervention 1.8-fold more than those who received tailored BBTI, which is consistent with results from a 2019 randomized clinical trial.^24^

In the primary outcomes, tailored BBTI significantly and greatly improved both insomnia severity and social disabilities at 1 and 3 months after the intervention compared with the waiting list control group. In particular, it is noteworthy that the large effect of 2 weeks of tailored BBTI on insomnia severity and daytime dysfunction after the intervention is similar to the findings of other studies on digitally delivered CBTI^18^ and face-to-face CBTI^13^ for 4 to 8 weeks. These findings suggest that a tailored intervention based on the stepped-care model can maximize the effect of CBTI, that the effect is sustained after only 2 weeks, and that this may be an effective prophylactic treatment for chronic insomnia disorder.

The effects of standard BBTI and self-monitoring on primary outcomes were also medium to large at the 3-month follow-up compared with the waiting list. Out of these, the effect sizes within each group showed a similar change between tailored and standard BBTI. These findings are consistent with a 2012 study^23^ of internet-delivered CBT for depression. That study found that standard CBT was effective for participants with mild depression but that tailored CBT could also be effective for those with mild to severe depression. Since the participants of our study had mild insomnia, they received a tailored or standard BBTI or self-monitoring interventions that could theoretically improve insomnia symptoms. However, improvements in insomnia severity at the 1-month follow-up were confirmed only in the tailored BBTI group. This suggests that tailored BBTI is a quicker-acting intervention than standard BBTI, which is the first time this result has been reported for tailored digitally delivered CBT, to our knowledge.

In the secondary outcomes of dysfunctional beliefs and sleep reactivity, the tailored BBTI group had statistically significant medium to large improvements in both outcomes at 3 months after the intervention compared with the self-monitoring and waiting list groups. Remarkably, in the FIRST assessment, while the participants who underwent tailored BBTI showed statistically significant moderate improvements with sleep reactivity, dysfunctional beliefs were significantly reduced in both tailored and standard BBTI groups. It has been shown that dysfunctional beliefs and sleep reactivity serve as risk factors for maintaining insomnia^32,33^ and that sleep reactivity works as a marker for increased risk of insomnia and as a risk factor for the onset of depression.^34^ Furthermore, worker productivity significantly and greatly improved in the tailored BBTI group compared with the standard BBTI group at 3 months after the intervention. The effects of tailored BBTI on sleep reactivity and productivity, as well as on other insomnia-related symptoms, suggest that a 2-week program of tailored BBTI for workers with insomnia is effective for improving sleep and enhancing productivity. Therefore, it is advantageous in terms of both primary and secondary prevention to provide an application for individually tailored BBTI to workers with insomnia.

### Limitations

This study has some limitations. First, the participants of this trial were all business workers aged approximately 40 years who had been categorized as experiencing insomnia based on the results of a previous study.^22^ According to the stepped-care model, digitally delivered CBTI is also effective for young adults and those with better sleep.^22^ In future research, it would be informative to examine the effect of tailored BBTI on insomnia-related symptoms among people with insomnia aged 20 to 30 years. In addition, the intensity of work an individual performs (eg, light office work vs heavy manual labor) might influence the individual’s quality of sleep, although we excluded shift workers and the individuals who had higher risk of causing serious harm from sleep loss (eg, individuals who operate heavy machinery). Second, there was a high dropout rate after 3 months. In particular, it was higher in both of the BBTI interventions. The reason for this is not clear from our study design. It would be informative to investigate the reasons for high dropout rates during follow-up periods and the behaviors of people who are prone to dropout in future research.

## Conclusions

This randomized clinical trial found that an application that provided tailored BBTI based on the stepped-care model quickly and greatly improved insomnia severity and social disabilities after just 2 weeks of use. In addition, the intervention successfully decreased the factors associated with developing and maintaining insomnia, but greater long-term follow-up is necessary. Furthermore, the tailored BBTI intervention resulted in enhanced productivity. Therefore, we conclude that it is advantageous for both primary and secondary prevention to provide an application for individually tailored BBTI to workers with insomnia. In future studies, reasons for dropout during the follow-up period should be investigated.

## Notes

Supplement 1. **Trial Protocol**

Supplement 2. **eTable 1.** Challenge Tasks Provided for Participants in the Tailored Brief Behavioral Therapy for Insomnia Group **eTable 2.** Outcome Measures in Each Group **eFigure 1.** The Changes in the Subscale for Sheehan Disability Scale Over Time for Each Group **eFigure 2.** Plots of Effect Sizes Over Time for the Subscale of Sheehan Disability Scale Between Groups **eFigure 3.** Plots of Effect Sizes for the Subscale of Sheehan Disability Scale Within Each Group

Supplement 3. **Data Sharing Statement**
