# Supplementary material for: Effects of a Tailored Brief Behavioral Therapy Application on Insomnia Severity and Social Disabilities Among Workers With Insomnia in Japan: A Randomized Clinical Trial
Source: JAMA Netw Open. 2020 Apr 14;3(4):e202775. doi: 10.1001/jamanetworkopen.2020.2775 (PMC7156995; doi:10.1001/jamanetworkopen.2020.2775)
Supplement: Supplement 1. — Trial Protocol [file jamanetwopen-3-e202775-s001.pdf]

Study Protocol and Statistical Analysis Plan

Basic Information:

Study Title: A tailored brief behavioral therapy app for insomnia: a single-blind randomized controlled trial

Region: Japan

Condition: Adult workers with insomnia

Date of protocol fixation: March 28, 2017

Actual trial start date: September 21, 2017

Study completion Date: February 23, 2018

Date analysis concluded: February 22, 2019

Background:

According to the stepped-care model, the effect size for using a cognitive behavioral therapy intervention for insomnia by utilizing information and communication technology (I-CBTI) is medium to large. However, since some interventions for I-CBTI require expert support to enhance remission rates, it has been reported that fully-automated use of an I-CBTI app without expert support has a high dropout rate.

Objective:

The aim of this study was to examine the effect of using a fully-automated and individually tailored brief behavior therapy app for insomnia (BBTI) for 2 weeks for workers with insomnia on insomnia-related symptoms, social disabilities, and work productivity.

Assessment Measures:

Primary outcomes: Insomnia Severity Index (ISI), Sheehan Disability Scale (SDISS)

Secondary outcomes: Dysfunctional beliefs and Attitudes about sleep Scale (DBAS), Ford Insomnia Response to Stress Test (FIRST), Work Limitation Questionnaire (WLQ)

Participants were assessed using the following scales over the internet pre-intervention, post-intervention (2-week), and with follow-ups at 1-month (FU1) and 3-months (FU3) post-intervention.

Study Design:

(1) Basic design: Parallel

(2) Randomization: Randomized

(3) Randomization unit: Individual

(4) Blinding: Single blind-investigators and assessors are blinded

(5) Control: self-monitoring (active control), wait-list (no treatment)

(6) Stratification: Yes

(7) Blocking: Yes

(8) Concealment: Central registration

Eligibility:

Inclusion criteria:

(1) workers aged 20 years or older

(2) total score in the Insomnia Severity Index (ISI) of ≥ 8

(3) complaints of initial, middle, and/or terminal insomnia

(4) performing work during the day

Exclusion criteria:

(1) having a medical history of posttraumatic stress disorder, depressive disorders or sleep disorders without insomnia

(2) reporting suicidal ideation

(3) receiving pharmacological and/or psychological treatments

(4) shift-workers

(5) likelihood of causing serious risk from sleep loss (e.g., traffic accident).

Sample size:

Sample size was based on a power analysis conducted for the ISI scores. Effect sizes were estimated from 2-weeks of BBTI pilot data from our group acquired prior to this study (Hedges' g in the ISI scores from pre-intervention [Mean = 12.38, SD = 3.57] to 1-month follow-up [Mean = 8.48, SD = 3.41] was 1.10 in 21 workers with insomnia. With power of 0.9 to detect a significant difference at p = 0.05 (2-sided), it was calculated that 12 persons would be required for each group. Since the dropout rate for self-help CBTI is reportedly highly variable between studies30, we aimed to allow for a 40% dropout rate which would require 20 participants to be recruited per group.

Interventions

Participants downloaded the app for sleep improvement developed for this study to their android smartphone or iPhone (Table 1). They were each conducted in one of the following groups.

Tailored BBTI

Participants were assessed for sleep-related daily habits (e.g., bed/wake time, working, exposure to bright-light) before intervention. In addition, we prepared 26 challenge tasks based on the results of assessment (Figure 1). For each challenge task, the difficulty and effect levels ranged from one star (very easy/low effect) to five stars (very difficult/high effect). The tasks were categorized within various CBT-I techniques: sleep hygiene (24 tasks), sleep scheduling (4 tasks), or relaxation (2 tasks) (Figure 1). For example, an individual who had complained of difficulty initially getting to sleep would be suggested the challenge task "Getting out of bed when unable to sleep, and going to bed only when sleepy" based on the stimulus control technique (difficulty level: ★★★★★, effect level: ★★★★★).

Participants chose 1 to 3 of the tasks which had been suggested to them, then they focused on these tasks for 2 weeks. They estimated whether each task was implemented or not day-to-day, and recorded daily sleep conditions including bed/wake times, sleep onset latency, wake after sleep onset, nap and vitality of the day by using a sleep diary. Messages sent through the app to participants every day during the 2 weeks to remind participants to keep recording the sleep diary. When the task was not conducted often enough, instructions to rethink the task were presented with several specific examples on the 3rd and 10th day. For example, when it was difficult to conduct the challenge task "Getting out of bed when unable to sleep, and going to bed only when sleepy", participants were provided with suggestions for what to do, such as not checking the clock if they awoke in the night. Feedback on the state of sleep and the achievement status of the task from the previous day were provided on a daily basis. To motivate them to conduct the challenge tasks, a picture of the face of an expert of sleep scientist (I.O.) provided instructions and feedback comments. Furthermore, an article about sleep sciences (e.g., about the relationship between the circadian rhythm and bright-lights) along with individually tailored challenge tasks were delivered every day.

Table 1. The details of the trial

	TBBTI	SBBTI	SM	WL
Pre-intervention				
Measuring primary outcomes	●	●	●	●
Secondary outcomes	●	●	●	●
Assessment of sleep condition	●	-	-	-
Setting the challenging tasks	● (individually)	● (standard)	-	-
During the intervention				
Recoding sleep diaries	●	●	●	-
Recoding of the tasks conducted	●	-	-	-
Delivering an article about sleep sciences	● (individually)	● (standard)	● (standard)	-
Feedback on the state of sleep	●(daily; weekly)	● (weekly)	-	-
Feedback on the achievement status of the task	● (daily)	-	-	-
Post-intervention/1- and 3-month FU				
Primary outcomes	●	●	●	●
Secondary outcomes	●	●	●	●

Note. FU = follow-up. SBBTI = standard brief behavior therapy for insomnia. SM = self-monitoring. TBBTI = tailored BBTI. WL = wait-list control.

Figure 1. Challenge tasks provided for participants in tailored BBTI

	Category	Challenge tasks	Difficulty level	Effect level
1	SS	Setting of regularly sleep window (go-to-bad/get-out-of-bed time)	★★★★★	★★★★★
2	SS	Getting out of bed when unable to sleep/going to bed only when sleepy	★★★★★	★★★★★
3	SS	Setting time regularly getting out of bed on weekday and weekend	★★★	★★★★
4	SS	Getting up early (≤ 10 minutes) when wake up in the morning	★★★★	★★★★
5	RT	Conducting progressive muscle relaxation before sleep every night	★★★★	★★★★
6	RT	Conducting breathing relaxation before sleep every night	★	★★
7	SHE	Getting up in the morning, opening curtain and taking a sunlight	★★	★★★★
8	SHE	Taking a sunlight while eating the breakfast in a room	★★	★★★★
9	SHE	Taking a sunlight while walking to go to work	★★★	★★★★
10	SHE	Taking a sunlight by making time to spend on the balcony and/or beside the window	★★★	★★★★
11	SHE	Taking a sunlight while getting ready to go to work/leisure beside the window	★★	★★★★

12	SHE	Taking a sunlight while walking or airing in the morning	★★★★★	★★★★★
13	SHE	Do not use your mobile phone or smartphone for a prolonged time before going to sleep	★★★	★★★★
14	SHE	Do not use your PC or tablet before going to sleep	★★★★	★★★★
15	SHE	Do not watch TV before going to sleep	★★★★	★★★★
16	SHE	Dimming the light in your room an hour before going to sleep	★★	★★★
17	SHE	Relatively spending in a dim room before going to bed	★★	★★★
18	SHE	Do not perform work/household duties just before going to bed	★★	★★★
19	SHE	Do not go out of bright place (e.g., convenience store) in the night.	★★	★★★★
20	SHE	Taking a bath 1 to 2 hours before going to bed	★★	★★★★
21	SHE	Do not relax on the sofa or have daytime nap for prolonged time	★★★	★★★
22	SHE	Do not sit on the seat in bus or train. Do not have nap when sitting.	★★★★	★★★★
23	SHE	Stop drinking four hours before bedtime	★★★	★★★
24	SHE	Do not have a nightcap before going to bed or when arousal during sleep	★★★	★★★
25	SHE	Do not drink or eat caffeine-containing product 6 hours before bedtime	★★★	★★★

26	SHE	Do not have a smoke an hour before going to bed	★★	★

Note. BBTI = brief behavior therapy for insomnia. RT = Relaxation Techniques. SS = sleep scheduling. SHE = sleep hygiene education.

Standard BBTI

Participants assigned to the standard BBTI group were delivered tasks relating to sleep hygiene for Week 1. They could view articles about sleep sciences (e.g., the mechanisms of sleep homeostasis and circadian rhythms) at any time, although one column about sleep sciences was delivered every day. For Week 2, explanations about sleep scheduling and relaxation techniques were delivered and some tasks were suggested. Participants pursued all suggested tasks and recorded a sleep diary for 2 weeks. On 7th and 14th day, feedback on sleep and implementation status was provided for participants.

Self-monitoring

Participants assigned to the self-monitoring group recorded a sleep diary for 2 weeks. In addition, they could view all articles about sleep sciences at any time, although one column about sleep science was delivered every day. After taking the questionnaires at the 3-month follow-up, they were allowed to enroll on a program of tailored BBTI.

Wait-list control

Participants assigned to the wait-list control group were only asked to complete questionnaires pre-intervention, post-intervention, and at the 1-month and 3-month follow-up periods. After completing the questionnaires at the 3-month follow-up, they were allowed to enroll on a program of tailored BBTI.

Data management and statistical analysis:

Most analyses will be based on the intent-to-treat (ITT) model. To examine the effect of tailored BBTI on insomnia-related symptoms and productivity, a mixed-effects model for repeated measures, which compensates for missing data, was used to compare pre-intervention, post-intervention and FU1, and FU3 data in all groups. When an interaction effect was shown, we conducted the Holm's post-hoc multiple comparison test.

In addition, we will estimate the effect sizes of scales within and between groups by using Hedges'g (95% CI). The effect sizes of all scales within groups were computed as follows: at pre-intervention vs. post-intervention, at pre-intervention vs. FU1, and pre-intervention vs. FU3 in each group. Effect sizes between groups were analyzed in each period (at post-intervention, FU1, and FU3, respectively) after calculating change score of each scale (*⊿*) from pre-intervention to each period (e.g., tailored BBTI vs. standard BBTI for score of *⊿*ISI at post-intervention). To compare dropout rate between groups, we conducted χ^2 tests.
