# Supplementary material for: Effects of a Tailored Brief Behavioral Therapy Application on Insomnia Severity and Social Disabilities Among Workers With Insomnia in Japan: A Randomized Clinical Trial
Source: JAMA Netw Open. 2020 Apr 14;3(4):e202775. doi: 10.1001/jamanetworkopen.2020.2775 (PMC7156995; doi:10.1001/jamanetworkopen.2020.2775)
Supplement: Supplement 2. — eTable 1. Challenge Tasks Provided for Participants in the Tailored Brief Behavioral Therapy for Insomnia Group eTable 2. Outcome Measures in Each Group eFigure 1. The Changes in the Subscale for Sheehan Disability Scale Over Time for Each Group eFigure 2. Plots of Effect Sizes Over Time for the Subscale of Sheehan Disability Scale Between Groups eFigure 3. Plots of Effect Sizes for the Subscale of Sheehan Disability Scale Within Each Group [file jamanetwopen-3-e202775-s002.pdf]

Supplementary Online Content

Okajima I, Akitomi J, Kajiyama I, Ishii M, Murakami H, Yamaguchi M. Effects of a tailored brief behavioral therapy application on insomnia severity and social disabilities among workers with insomnia in Japan: a randomized clinical trial. *JAMA Netw Open*. 2020;3(4):e202775. doi:10.1001/jamanetworkopen.2020.2775

eTable 1. Challenge Tasks Provided for Participants in the Tailored Brief Behavioral Therapy for Insomnia Group

eTable 2. Outcome Measures in Each Group

eFigure 1. The Changes in the Subscale for Sheehan Disability Scale Over Time for Each Group

eFigure 2. Plots of Effect Sizes Over Time for the Subscale of Sheehan Disability Scale Between Groups

eFigure 3. Plots of Effect Sizes for the Subscale of Sheehan Disability Scale Within Each Group

This supplementary material has been provided by the authors to give readers additional information about their work.

© 2020 Okajima I et al. *JAMA Network Open.*

eTable 1. Challenge Tasks Provided for Participants in the Tailored Brief Behavioral Therapy for Insomnia Group

	**Category**	**Challenge tasks**	**Difficulty level**	**Effect level**
1	SS	Setting of regularly sleep window (go-to-bad/get-out-of-bed time)	★★★★★	★★★★★
2	SS	Getting out of bed when unable to sleep/going to bed only when sleepy	★★★★★	★★★★★
3	SS	Setting time regularly getting out of bed on weekday and weekend	★★★	★★★★
4	SS	Getting up early (≤ 10 minutes) when wake up in the morning	★★★★	★★★★
5	RT	Conducting progressive muscle relaxation before sleep every night	★★★★	★★★★
6	RT	Conducting breathing relaxation before sleep every night	★	★★
7	SHE	Getting up in the morning, opening curtain and taking a sunlight	★★	★★★★
8	SHE	Taking a sunlight while eating the breakfast in a room	★★	★★★★
9	SHE	Taking a sunlight while walking to go to work	★★★	★★★★
10	SHE	Taking a sunlight by making time to spend on the balcony and/or beside the window	★★★	★★★★
11	SHE	Taking a sunlight while getting ready to go to work/leisure beside the window	★★	★★★★
12	SHE	Taking a sunlight while walking or airing in the morning	★★★★★	★★★★★
13	SHE	Do not use your mobile phone or smartphone for a prolonged time before going to sleep	★★★	★★★★

© 2020 Okajima I et al. *JAMA Network Open.*

14	SHE	Do not use your PC or tablet before going to sleep	★★★★	★★★★
15	SHE	Do not watch TV before going to sleep	★★★★	★★★★
16	SHE	Dimming the light in your room an hour before going to sleep	★★	★★★
17	SHE	Relatively spending in a dim room before going to bed	★★	★★★
18	SHE	Do not perform work/household duties just before going to bed	★★	★★★
19	SHE	Do not go out of bright place (e.g., convenience store) in the night.	★★	★★★★
20	SHE	Taking a bath 1 to 2 hours before going to bed	★★	★★★★
21	SHE	Do not relax on the sofa or have daytime nap for prolonged time	★★★	★★★
22	SHE	Do not sit on the seat in bus or train. Do not have nap when sitting.	★★★★	★★★★
23	SHE	Stop drinking four hours before bedtime	★★★	★★★
24	SHE	Do not have a nightcap before going to bed or when arousal during sleep	★★★	★★★
25	SHE	Do not drink or eat caffeine-containing product 6 hours before bedtime	★★★	★★★
26	SHE	Do not have a smoke an hour before going to bed	★★	★

Note. bBTi = brief behavior therapy for insomnia. RT = Relaxation Techniques. SS = sleep scheduling. SHE = sleep hygiene education.

© 2020 Okajima I et al. *JAMA Network Open.*

eTable 2. Outcome Measures in Each Group

	Tailored BBTI (n=24)	Standard BBTI (n=23)	SM (n=23)	WL (n=22)	Time effect (df = 3, 88)	Interaction effect (df = 9, 264)
Insomnia severity; ISI (rage: 0[best]-28[worst])					F = 25.48, p < 0.01, η_G^2 = 0.09	F = 5.25, p < 0.01, η_G^2 = 0.06
pre-intervention	12.13 (3.80)	11.17 (3.19)	10.30 (3.88)	11.68 (4.17)		
post-intervention	9.54 (3.68)	10.17 (3.74)	10.48 (3.58)	10.18 (4.20)		
1-month follow-up	7.21 (3.71)	9.04 (5.02)	8.91 (3.60)	10.64 (4.23)		
3-month follow-up	6.46 (1.69)	6.83 (3.13)	8.26 (4.50)	11.50 (4.00)		
Disability in social life; SDISS-social (rage: 0[best]-10[worst])					F = 7.57, p < 0.01, η_G^2 = 0.03	F = 6.03, p < 0.01, η_G^2 = 0.07
pre-intervention	2.75 (2.15)	3.09 (2.17)	2.61 (1.78)	2.64 (2.42)		
post-intervention	2.25 (2.21)	3.04 (2.12)	2.04 (1.66)	2.00 (2.18)		
1-month follow-up	1.83 (1.55)	1.57 (1.78)	1.96 (1.77)	2.41 (1.97)		
3-month follow-up	0.58 (1.10)	1.39 (1.62)	2.61 (2.23)	3.23 (2.60)		
Disability on work performance; SDISS-work (rage: 0[best]-10[worst])					F = 8.94, p < 0.01, η_G^2 = 0.04	F = 3.31, p < 0.01, η_G^2 = 0.04
pre-intervention	3.04 (2.03)	3.04 (1.92)	3.17 (1.90)	2.77 (1.97)		
post-	2.25	2.74	2.65	2.32		

© 2020 Okajima I et al. *JAMA Network Open.*

intervention	(1.54)	(1.79)	(1.72)	(2.10)		
1-month follow-up	2.29 (1.83)	1.74 (1.45)	2.00 (2.00)	2.86 (1.73)		
3-month follow-up	1.25 (1.11)	1.83 (1.11)	2.35 (2.44)	3.09 (2.11)		
Disability on family life; SDISS-family (rage: 0[best]-10[worst])					F = 3.67, p = 0.01, η_G^2 = 0.02	F = 1.74, p = 0.08, η_G^2 = 0.02
pre-intervention	1.67 (1.69)	2.17 (2.27)	2.22 (2.24)	2.05 (2.50)		
post-intervention	1.29 (1.49)	1.65 (1.97)	1.91 (2.15)	1.64 (2.06)		
1-month follow-up	1.38 (1.38)	1.83 (1.72)	2.48 (2.06)	1.95 (2.32)		
3-month follow-up	0.46 (0.66)	1.22 (1.70)	1.30 (1.87)	2.55 (3.25)		
Sleep reactivity; FIRST (rage: 1[best]-36[worst])					F = 6.12, p < 0.01, η_G^2 = 0.02	F = 3.18, p < 0.01, η_G^2 = 0.04
pre-intervention	21.08 (4.94)	20.87 (4.67)	20.00 (5.78)	21.91 (4.71)		
post-intervention	22.88 (3.95)	22.09 (4.63)	20.04 (5.00)	23.05 (4.59)		
1-month follow-up	19.04 (6.96)	20.13 (4.43)	19.78 (3.12)	22.09 (4.78)		
3-month follow-up	17.29 (3.18)	22.04 (5.69)	19.70 (4.35)	21.77 (4.82)		
Dysfunctional beliefs and attitudes; DBAS (rage: 0[better]-160[worse])					F = 3.46, p = 0.02, η_G^2 = 0.01	F = 4.23, p < 0.01, η_G^2 = 0.04
pre-intervention	74.63 (24.29)	74.96 (23.02)	70.57 (23.97)	78.32 (21.31)		
post-	68.00	70.91	69.04	79.64		

© 2020 Okajima I et al. *JAMA Network Open.*

intervention	(21.35)	(25.88)	(17.44)	(24.96)		
1-month follow-up	75.13 (32.59)	71.61 (16.29)	67.13 (15.43)	80.09 (22.28)		
3-month follow-up	52.83 (24.82)	62.91 (21.48)	72.43 (24.01)	84.50 (28.42)		
Worker productivity (presenteeism); WLQ （range: 0[worse]-100[better]）					F = 3.99, p = 0.01, η_G^2 = 0.01	F = 3.03, p < 0.01, η_G^2 = 0.03
pre-intervention	92.70 (3.63)	92.12 (3.14)	92.76 (4.15)	93.30 (2.91)		
post-intervention	93.89 (3.88)	94.10 (3.70)	93.52 (3.78)	93.85 (3.72)		
1-month follow-up	96.09 (3.37)	92.80 (4.18)	92.83 (4.58)	93.57 (5.05)		
3-month follow-up	95.43 (3.72)	91.94 (3.59)	92.73 (3.64)	94.16 (4.17)		

Note. BBTI = brief behavior therapy for insomnia. DBAS = Dysfunctional Beliefs and Attitudes about Sleep. F = female. FIRST = Ford Insomnia Response to Stress Test. ISI = Insomnia Severity Index. M = Male. SDISS = Sheehan Disability Scale. WLQ = Work Limitation Questionnaire.

Standard deviations are shown in parentheses.

© 2020 Okajima I et al. *JAMA Network Open.*

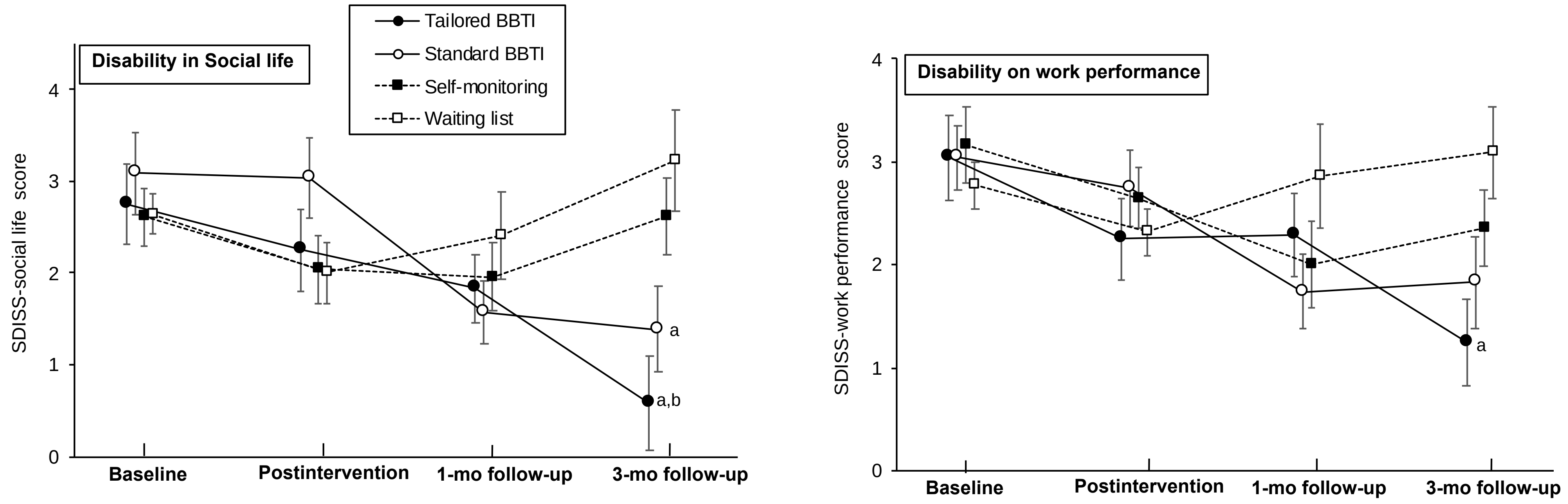

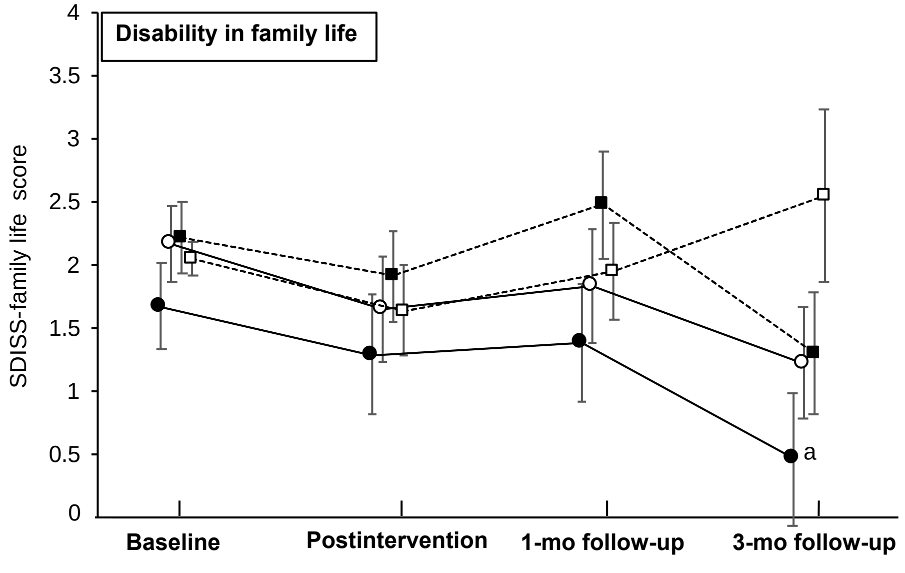

eFigure 1. Changes in Outcome Measures Through Time for Each Group

Error bars indicate SE; BBTI, brief behavioral therapy for insomnia; SDISS, Sheehan Disability Scale.

[a] Statistically significantly different compared with the waiting list group.

[b] Statistically significantly different compared with the self-monitoring group.

© 2020 Okajima I et al. *JAMA Network Open.*

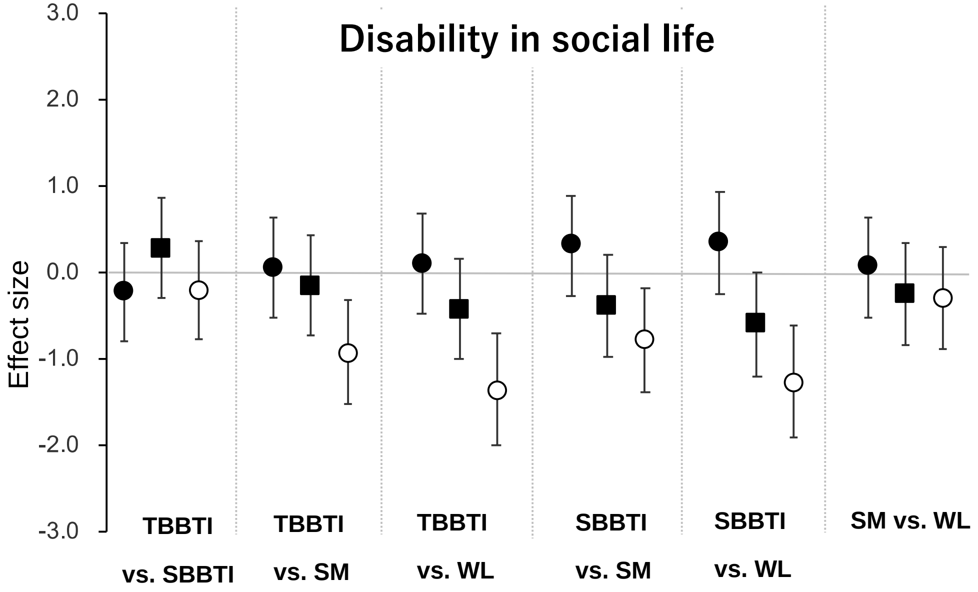

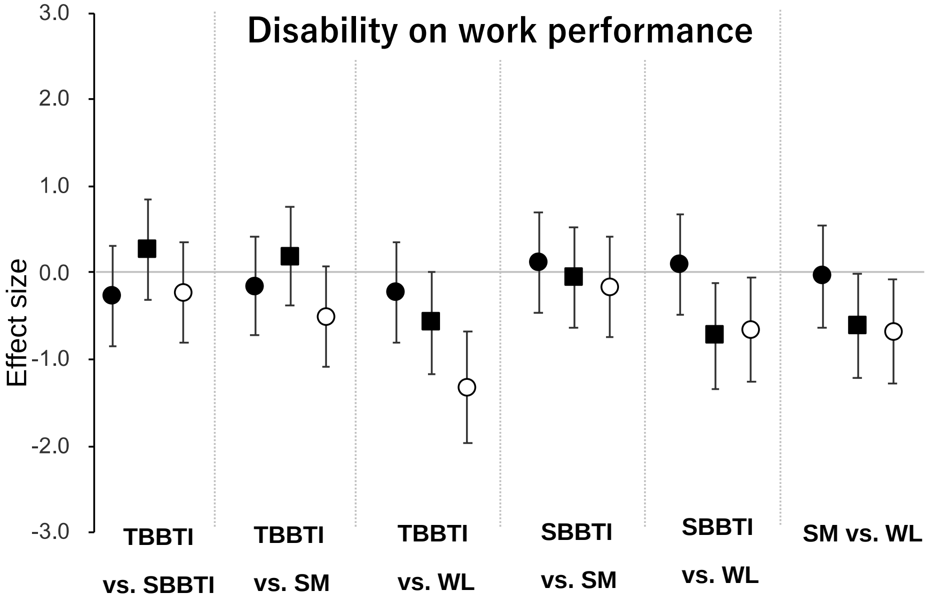

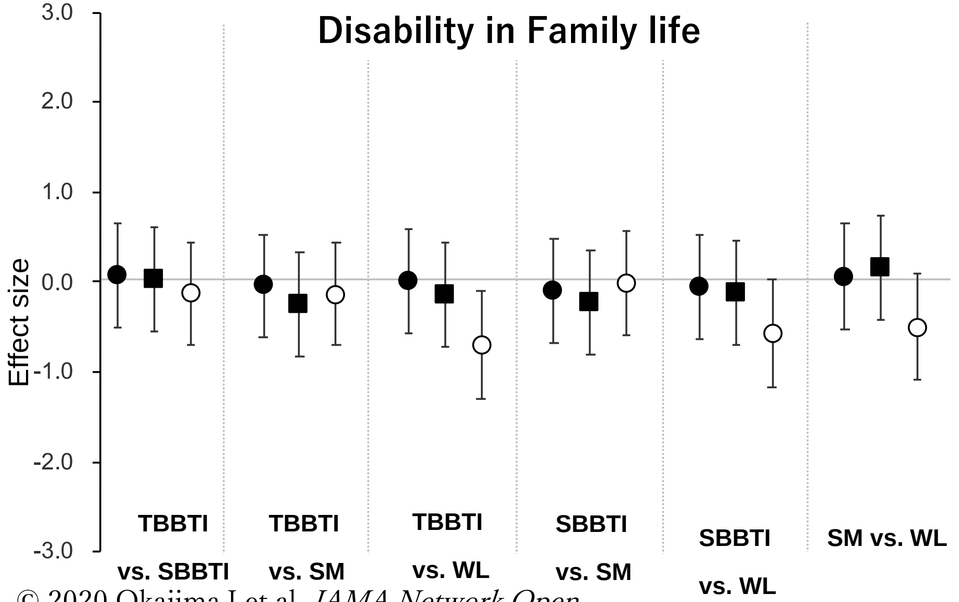

eFigure 2. Plots of Effect Sizes Through Time for Each Outcome Measure Between Groups

Error bars indicate 95% CI; SBBTI, standard brief behavioral therapy for insomnia; SM, self-monitoring; TBBTI, tailored brief behavioral therapy for insomnia; and WL, waiting list.

© 2020 Okajima I et al. *JAMA Network Open.*

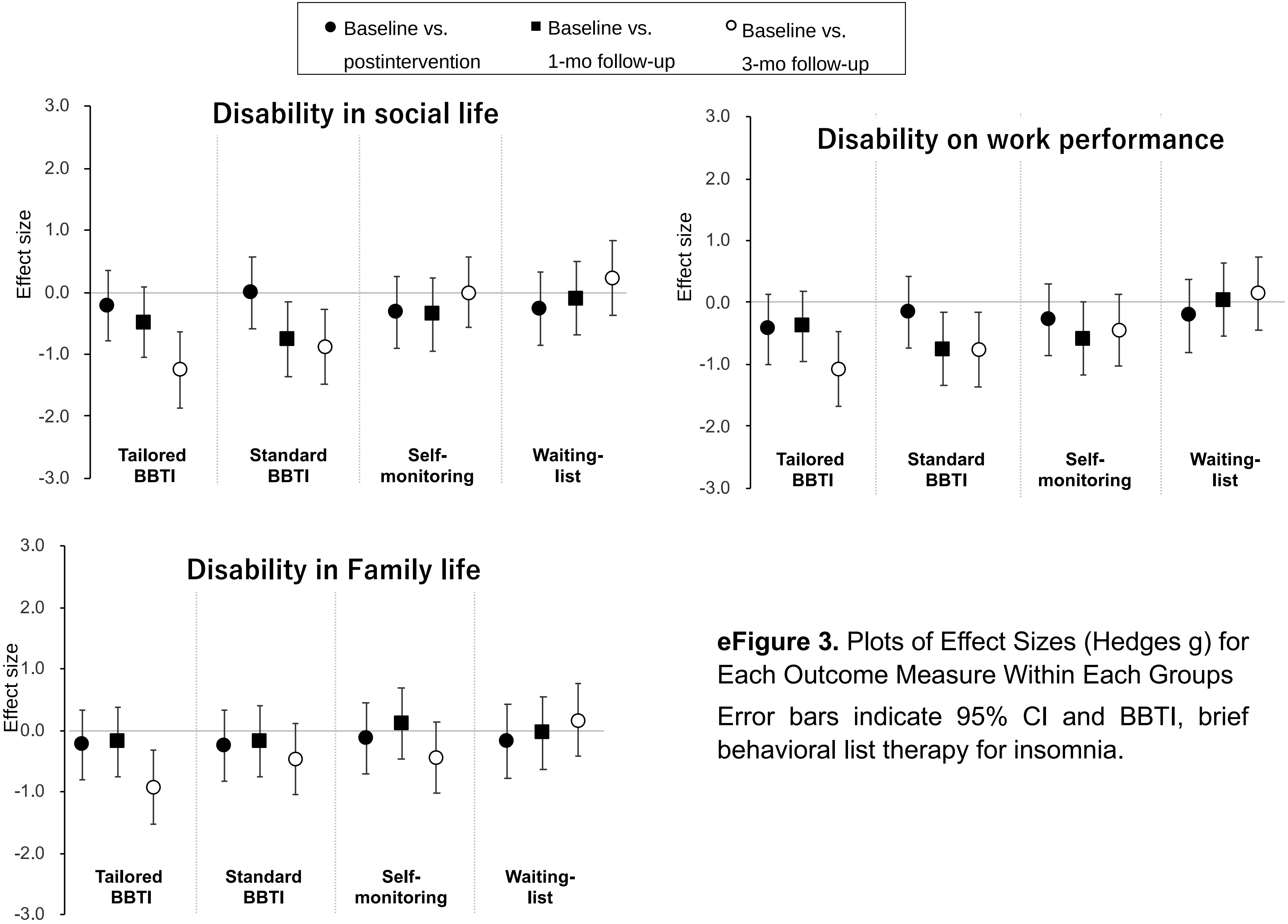

eFigure 3. Plots of Effect Sizes (Hedges g) for Each Outcome Measure Within Each Groups

Error bars indicate 95% CI and BBTI, brief behavioral list therapy for insomnia.

© 2020 Okajima I et al. *JAMA Network Open.*
