# Supplementary material for: Effects of a Tailored Brief Behavioral Therapy Application on Insomnia Severity and Social Disabilities Among Workers With Insomnia in Japan: A Randomized Clinical Trial
Source: JAMA Netw Open. 2020 Apr 14;3(4):e202775. doi: 10.1001/jamanetworkopen.2020.2775 (PMC7156995; doi:10.1001/jamanetworkopen.2020.2775)
Supplement: Supplement 3. — Data Sharing Statement [file jamanetwopen-3-e202775-s003.pdf]

Data Sharing Statement

Okajima. Effects of a Tailored Brief Behavioral Therapy Application on Insomnia Severity and Social Disabilities Among Workers With Insomnia in Japan. *JAMA Netw Open*. Published April 14, 2020.
10.1001/jamanetworkopen.2020.2775

Data
Data available: No
